# Prosecution of Elder Neglect: A Close Examination of Cases From King County, Washington

**DOI:** 10.1093/geroni/igaa057.067

**Published:** 2020-12-16

**Authors:** Kriti Gogia, Alyssa Elman, Sunday Clark, Page Ulrey, Marie-Therese Connolly, Elizabeth Bloemen, Billie Johnsson, Tony Rosen

**Affiliations:** 1 Weill Cornell Medicine, New York, New York, United States; 2 King County Prosecuting Attorney's Office, Seattle, Washington, United States; 3 University of Southern California Leonard Davis School of Gerontology, Los Angeles, California, United States; 4 University of Colorado School of Medicine, Aurora, Colorado, United States; 5 NewYork-Presbyterian Hospital, New York, New York, United States

## Abstract

Elder neglect is common and can have catastrophic consequences. Cases may benefit from integrated responses from multiple sectors. Little research exists describing prosecutorial involvement and its impact, but existing evidence suggests neglect is seldom criminally prosecuted. Our goal was to closely examine neglect prosecution in a jurisdiction that has been a leader in using prosecution to attempt to address it. We quantitatively and qualitatively analyzed legal case files of felony elder neglect prosecuted in King County, Washington from 2008-2011. 13 cases were prosecuted, with a total of 10 victims. 90% of victims were female, with a median age of 88. 90% were unable to ambulate, and 90% had dementia. Defendants were commonly the victim’s adult child (38%). 23% had previous criminal citations/convictions. 46% of cases occurred in an Adult Family Home. 15% of cases went to trial, and all trial cases ended in conviction of some charge. Themes identified included: (1) perpetrators were either professional caregivers receiving compensation or non-professional caregivers financially dependent on the victim, (2) victims were malnourished and severely injured at time of reporting, and (3) medical expert contribution is imperative given complexity of these cases. Victims were unable to participate in prosecution in any case. This research shows that these cases are seldom prosecuted, even in a jurisdiction focusing on this phenomenon, but highlights characteristics of cases and demonstrates they may be prosecuted without victim participation. Future research is needed to examine prosecution’s impact on elder neglect to better understand how it may be optimally used.

